# Colorectal Cancer Presenting with Constipation During Pregnancy

**DOI:** 10.7759/cureus.1190

**Published:** 2017-04-25

**Authors:** Adriana Jones, Michael R Povlow

**Affiliations:** 1 Medical Student, University of Central Florida College of Medicine

**Keywords:** colorectal cancer, constipation, pregnancy, bowel obstruction, colon/rectal cancers, metastatic colorectal cancer, cancer in pregnancy

## Abstract

Colorectal cancer is a rare occurrence during pregnancy and can present with symptoms that are common during pregnancy such as constipation.This can make the diagnosis of colorectal cancer during pregnancy difficult. Management of colorectal cancer during pregnancy is similar to the treatment of non-pregnant patients, but with fetal safety in mind.

This case report describes a 33-year-old female gravida two para one (G2P1) at 29 weeks gestation who presented with a complete bowel obstruction. Colonoscopy, magnetic resonance imaging (MRI) and later resection showed an obstructing malignancy which was then resected through an exploratory laparotomy with left hemicolectomy. Postoperatively, there was a concern for sepsis, so labor was induced and the baby was delivered vaginally. The patient then continued with chemotherapy with hematology-oncology.

High clinical suspicion is needed to diagnose colorectal cancer during pregnancy. Once diagnosed, surgery can be considered if resectable, taking into account gestational age. Fetal safety is a major consideration during treatment.

## Introduction

Colorectal cancer is the seventh most common type of cancer found in pregnancy, estimated to occur one in every 13,000 pregnancies [1]. Constipation is a common symptom experienced during pregnancy; however, constipation is also one of the earliest symptoms of colorectal cancer. Other common presenting symptoms of colorectal cancer include abdominal pain, vomiting, and anemia; all of which are commonly seen in pregnancy. This is a major reason why colorectal cancer may be diagnosed later in pregnancy. This allows more spread of cancer and makes it difficult to treat [1]. In the past, colorectal cancer has been uncommon during pregnancy, often due to the younger age of females who become pregnant. In more recent years and in the future, the incidence during pregnancy is expected to increase with more and more females delaying childbirth [2]. The purpose of this case report is to increase awareness about colorectal cancer in pregnant women given the similarity of symptoms between normal pregnancy and cancer, and to explain that the treatment of colorectal cancer during pregnancy is similar to when not pregnant, but with added caution for fetal safety [3]. Informed consent statement was obtained for this study.

## Case presentation

A 33-year-old female gravida two para one (G2P1) at 29 weeks gestation by last menstrual period presented to labor and delivery with nausea, vomiting, crampy abdominal pain, and no bowel movement for over two weeks. She denied blood per rectum. She had a history of chronic constipation. Family history is significant for grandfather having colon cancer. She was in the hospital for several days receiving a bowel regimen of laxatives and enemas that did not produce a bowel movement. She then underwent a colonoscopy before being transferred. 

Vitals on admission were temperature - 98.6 Farenheit, pulse - 88 beats per minute, and blood pressure (BP)- 125/70 mmHg. Pertinent labs on admission included a white blood cell count 4.52x10^9^/L, hemoglobin 10.0 g/dL, hematocrit 29.2%, mean corpuscular volume 89.6 *u*m^3^, blood urea nitrogen 2 mg/dL, creatinine .35 mg/dL, calcium 8.0 mg/dL, and albumin 3.0 g/dL.

**Figure 1:**
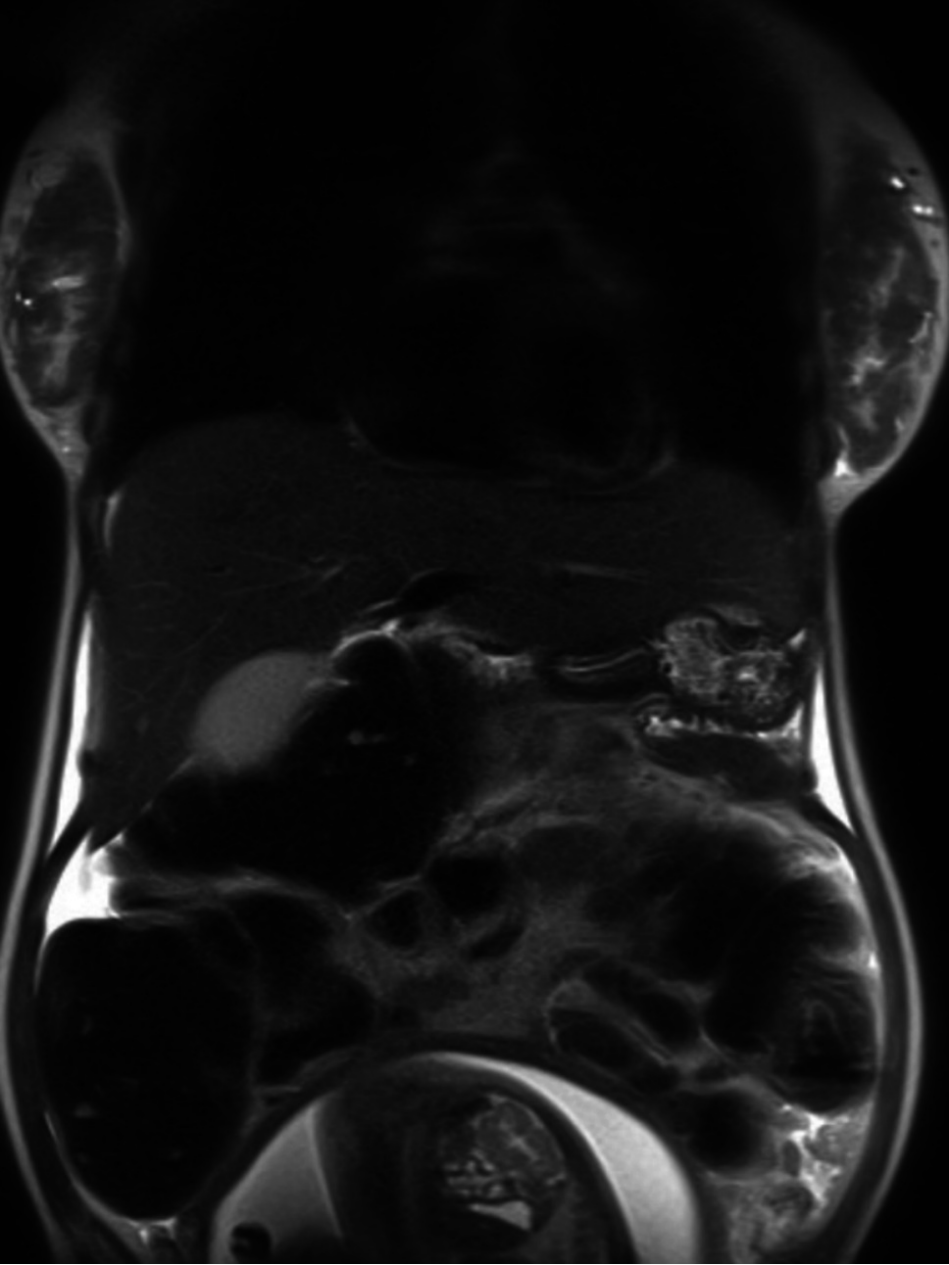
MRI of abdomen without contrast MRI showing obstruction of the distal colon with dilation of the upstream colon and retained feces. Gravid uterus is partially seen.

Colonoscopy of the mid or distal descending colon showed an obstructing mass. The mass completely occluded the lumen and was large and friable. Colonic obstruction with retained feces was confirmed on magnetic resonance imaging (MRI) (Figure 1).

One day later an exploratory laparotomy with left hemicolectomy and anastomosis was done with the obstetrics (OB) team present in the case of delivery. Resection showed moderately differentiated adenocarcinoma with extension to the visceral peritoneum and four of 17 lymph nodes.

After surgery, the patient was examined and was noted to be two centimeters dilated and 70% effaced. The patient received magnesium sulfate for neuroprotection of the fetus [4]. Four days post-operation, the patient started having abdominal pain, nausea, and emesis with regular contractions. She also had tachycardia, low-grade fever, and hypotension. Vitals were temperature - 99.4 Fahrenheit, pulse 128 beats per minute, respiratory rate 16 breaths per minute, and BP 90/66 mmHg. Due to concern for possible sepsis, labor was induced with Pitocin, antibiotics to cover group B strep, and magnesium for neuroprotection of fetus [4]. She delivered vaginally. The patient improved after delivery and blood cultures were negative. The patient was then referred to hematology-oncology to follow-up with chemotherapy for colon cancer.

## Discussion

This case report discusses a patient who presented with constipation, abdominal pain, nausea, and vomiting. Past reports have shown that common presenting symptoms of colorectal cancer can be attributed to normal pregnancy. These symptoms include nausea, vomiting, abdominal pain, constipation and anemia [1]. High clinical suspicion is needed to diagnose colorectal cancer during pregnancy when a patient is presenting with these symptoms. In this case, the patient had no bowel movement for two weeks with failed medical management of constipation, suggesting bowel obstruction.

When therapeutic intervention is necessary, endoscopy should be done for diagnosis in pregnant women with suspected colorectal cancer. It is the safe alternative to radiologic imaging during pregnancy in order to reduce radiation exposure to the fetus [5]. MRI is chosen as another mode of imaging during pregnancy in order to avoid ionizing radiation [6].

After diagnosis through endoscopy, surgery is a consideration. The recommendation for a resectable colorectal cancer during pregnancy is to resect cancer without disturbing the pregnancy prior to 20 weeks gestation. This allows the fetus to continue to grow and will allow cancer to be removed without further risk of metastasis. After 20 weeks of gestation, surgery can be postponed to allow for fetal lung maturity [7]. The patient should then be managed as a non-pregnant patient would be managed with fetal safety in mind. If maternal and/or fetal health were threatened, then pre-term delivery would be considered [3]. After delivery, the patient should be managed according to standard colorectal cancer guidelines based on grade and stage.

In this case, however, this patient had bowel obstruction by the malignant mass which increased the risk of perforation and sepsis. Therefore, she was taken urgently for exploratory laparotomy. Post-operatively, the patient and fetus’ safety was threatened by possible sepsis, so she delivered pre-term.

## Conclusions

Colorectal cancer is difficult to diagnose during pregnancy. Patients present with symptoms that can be attributed to normal pregnancy. Therefore, high clinical suspicion is imperative in order to investigate further with diagnostic tests. The safest intervention during pregnancy is endoscopy [5], MRI [6], and then surgery can be considered if the cancer is resectable. Pregnant patients are managed similar to non-pregnant patients, but with a special consideration for fetal safety. After delivery, patients can continue with normal colorectal treatment.
